# Melatonin Protects Rabbit Somatic Cell Nuclear Transfer (SCNT) Embryos from Electrofusion Damage

**DOI:** 10.1038/s41598-020-59161-6

**Published:** 2020-02-10

**Authors:** Pengxiang Qu, Chong Shen, Yue Du, Hongyu Qin, Shiwei Luo, Sixin Fu, Yue Dong, Shuaiqingying Guo, Fang Hu, Ying Xue, Enqi Liu

**Affiliations:** 1 Laboratory Animal Centre, Xi’an Jiaotong University Health Science Centre, Xi’an, Shaanxi 710061 China; 2 Research Institute of Atherosclerotic Disease, Xi’an Jiaotong University Cardiovascular Research Centre, Xi’an, Shaanxi 710061 China; 3 Nuffield Division of Clinical Laboratory Medicine, Radcliffe Department of Medicine, University of Oxford, Oxford, OX3 9DU UK

**Keywords:** Cloning, Reprogramming

## Abstract

The study’s objectives were to examine the effects of electrofusion on rabbit somatic cell nuclear transfer (SCNT) embryos, and to test melatonin as a protective agent against electrofusion damage to SCNT embryos. The levels of reactive oxygen species (ROS), the epigenetic state (H3K9me3), and the content of endoplasmic reticulum (ER) stress-associated transcripts (IRE-1 and CHOP) were measured. Melatonin was added during the preimplantation development period. The total blastocyst cell numbers were counted, and the fragmentation rate and apoptotic index were determined and used to assess embryonic development. Electrofusion increased (1) ROS levels at the 1-, 2-, 4-, and 8-cell stages; (2) H3K9me3 levels at the 2-, 4-, and 8-cell stage; and (3) the expression of IRE-1 and CHOP at the 8-cell, 16-cell, morula, and blastocyst stages. The treatment of SCNT embryos with melatonin significantly reduced the level of ROS and H3K9me3, and the expression levels of IRE-1 and CHOP. This treatment also significantly reduced the fragmentation rate and apoptotic index of blastocysts and increased their total cell number. In conclusion, the electrofusion of rabbit SCNT embryos induced oxidative stress, disturbed the epigenetic state, and caused ER stress, while melatonin reduced this damage. Our findings are of signal importance for improving the efficiency of SCNT and for optimizing the application of electrical stimulation in other biomedical areas.

## Introduction

Somatic cell nuclear transfer (SCNT) is important in generating genetically modified animals for improving breeding stock, modelling human diseases, and as a method of treatment with therapeutic potential^1^. Since the first cloned sheep Dolly was generated by SCNT in 1997, dozens of mammals have been successfully cloned over the past two decades^2^. However, SCNT technology still suffers from low efficiency, and this limits its application in agriculture and biomedicine^3^. Some factors, such as the cell cycle of oocytes and donor cells, the damage to reconstructed embryos, inferior culture medium, and incomplete reprogramming, have been identified as important factors affecting normal SCNT embryonic development^1,4,5^. The method used to fuse the donor cells and recipient oocytes is particularly important in determining the outcome of SCNT. There are several fusion methods. One is chemical fusion, which is generally toxic to embryos and thus rarely used^6,7^. Another is virus fusion using inactivated hemagglutinating virus of Japan (HVJ), however, the potential risk of HVJ to the embryo development remains unclear; the biological safety of this virus is questionable and the fusion rate is not especially high. Electrofusion is a method that has been widely used with a good success rate^8,9^. One benefit of using electrofusion is that the physical parameters of the technique can be fixed accurately and are easily reproducible. This can yield a higher fusion rate relative to other fusion^10^. The mechanism of electrofusion centers on the liquid flow model. When the plasmalemmas of donor and recipient cells contact each other under electrofusion, they generate micropores and the rearrangement of phospholipid molecules results in cell fusion^11^. Although electrostimulation is effective in many embryonic manipulations, the potential adverse effects on embryonic development are not well understood.

Previous studies reported that electrostimulation induced ROS in the embryos of rats, mice and pigs but only a few studies focused on SCNT embryos^12,13^. SCNT embryos can manifest higher ROS levels, and this can cause damage to mitochondria, cell membranes, and DNA, and abnormal gene expression^5,14,15^. Also, abnormal epigenetic reprogramming occurs in a large proportion of cloned embryos, and this impedes improvement in SCNT efficiency^1,9^. Electrostimulation also regulates the epigenetic state in somatic cells; however, there are few reports on the effect of electrostimulation on the epigenetic state of embryos^16,17^. In this study, we optimized the electrofusion procedure, and studied its effects on SCNT embryos, oxidative stress, epigenetic state and ER stress. Also, the developmental competence of SCNT embryos was assessed in terms of their fragmentation rate, apoptotic index, blastocyst rate, and total cell number in blastocysts.

## Results

### Rabbit SCNT embryos after electrofusion had higher cleavage and blastocyst rates

The reconstructed embryos and the cleavage embryos were observed and analyzed. There was no significant difference in reconstruction rate among the non-electrofusion group, the electrofusion group at 1.6 kV/cm, and the electrofusion group at 3.2 kV/cm, but the cleavage and blastocyst rates in the non-electrofusion group were significantly lower than in the electrofusion groups at 1.6 kV/cm and 3.2 kV/cm (*P* < 0.05) **(**Table 1**)**. There was no significant difference in cleavage and blastocyst rates between the groups undergoing electrofusion at 1.6 kV/cm and 3.2 kV/cm **(**Table 1**)**.

**Table 1:** Reconstructed rate, cleavage rate and blastocyst rate of cloned rabbit embryos in the non-electrofusion group and the electrofusion groups at 1.6 kV/cm and at 3.2 kV/cm.

Group	No. oocytes	Reconstructed rate (%)	Cleavage rate (%)	Blastocyst rate (%)
Non-electrofusion	206	183 (88.8)^a^	40 (21.8)^a^	6 (15.0)^a^
Electrofusion (1.6 kV/cm)	208	176 (84.6) ^a^	140 (79.5)^b^	46 (32.8)^b^
Electrofusion (3.2 kV/cm)	215	180 (82.6) ^a^	146 (81.1)^b^	44 (30.1)^b^

Different superscripts within same column indicate significant difference (P < 0.05).

### Electrofusion induced oxidative stress in rabbit SCNT embryo

**Figure 1:**
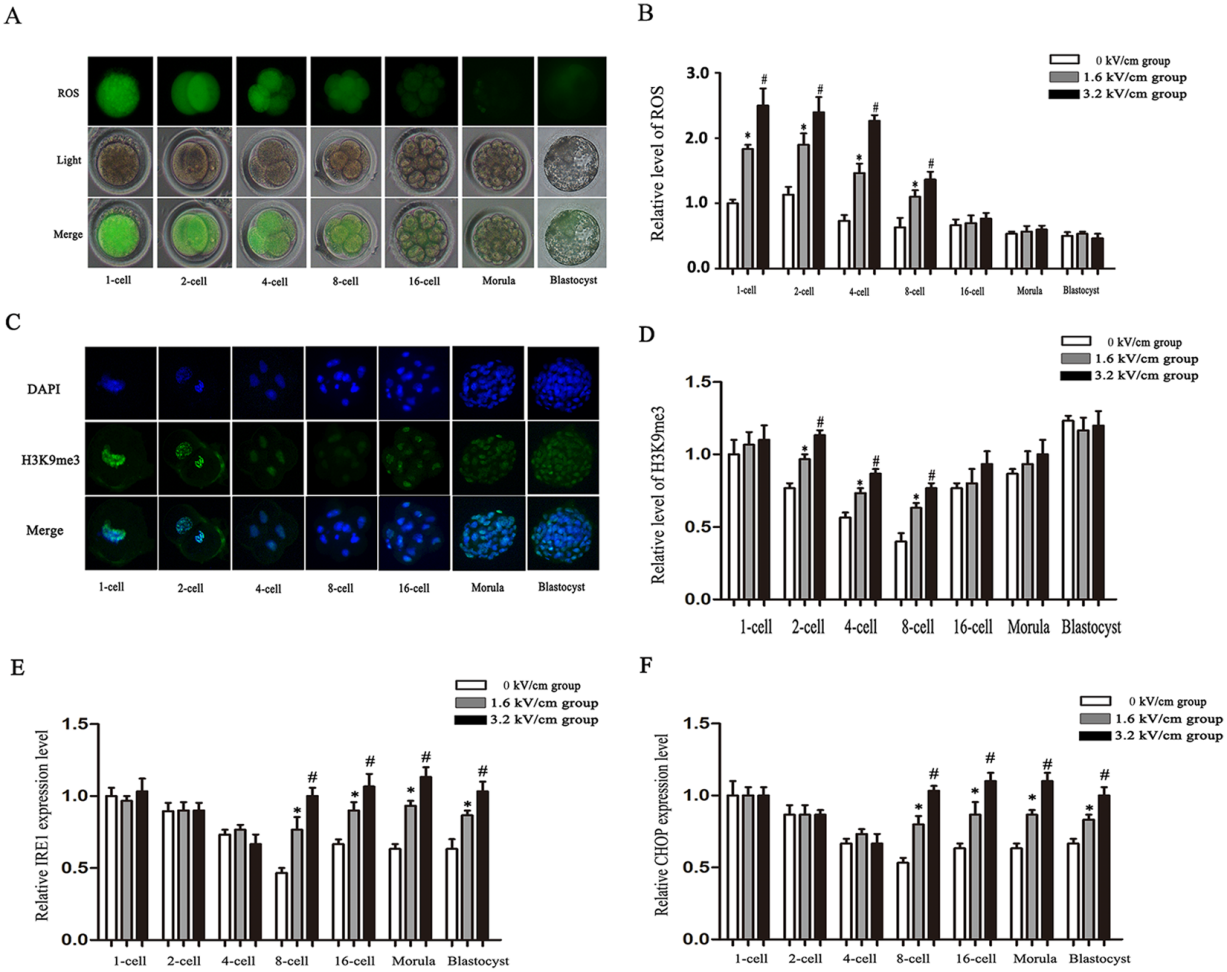
Effects of electrofusion on rabbit SCNT embryos. (**A**) A representative fluorescent image of rabbit SCNT embryos stained with dichlorodihydrofluorescein diacetate (DCFH-DA) assay for ROS production (green). The quantitation of ROS (**B**) in rabbit SCNT embryos at the indicated stages in non-electrofusion, and electrofusion at 1.6 kV/cm and 3.2 kV/cm, respectively. (**C**) A representative image of rabbit SCNT embryos stained with H3K9me3 (green) and DAPI for nuclei (blue). Relative levels of H3K9me3 (**D**) in rabbit SCNT embryos at the indicated stage in non-electrofusion, and electrofusion at 1.6 kV/cm and at 3.2 kV/cm, respectively. The relative expression levels of IRE1 (**E**) and CHOP (**F**) in rabbit SCNT embryos at the indicated stages in non-electrofusion, and electrofusion at 1.6 kV/cm and at 3.2 kV/cm, respectively. Different superscripts at the same stage in each group above the bars denote statistically significant differences (*P* < 0.05).

To measure the levels of ROS in SCNT embryos at the 1-, 2-, 4-, 8-, and 16-cell, morula and blastocyst stages, dichlorodihydrofluorescein diacetate (DCFH-DA) was used **(**Fig. 1A**)**. The results showed that ROS levels in electrically stimulated SCNT embryos at the 1-, 2-, 4-, and 8-cell stages were significantly higher than those in the embryos in the non-electrofusion group (*P* < 0.05) **(**Fig. 1B**)**. The level of ROS in the 3.2 kV/cm SCNT group was higher than that in the 1.6 kV/cm SCNT group (*P* < 0.05) (Fig. 1B). There were no significant differences of ROS levels in embryos at 16-cell, morula, and blastocyst stages among all groups (Fig. 1B).

### Electrofusion increased H3K9me3 levels in rabbit SCNT embryos

To detect the epigenetic states of SCNT embryos at the 1-, 2-, 4-, 8-, and 16-cell, morula, and blastocyst stages, the level of histone H3 lysine 9 trimethylation (H3K9me3) was assessed by immunofluorescence (Fig. 1C). The results showed that the levels of H3K9me3 in the electrically stimulated SCNT groups were significantly higher than those in the non-electrofusion group at the 2-, 4- and 8-cell stages (*P* < 0.05). The levels of H3K9me3 in the 3.2 kV/cm groups were significantly higher than those in the 1.6 kV/cm group at the 2-, 4- and 8-cell stages (*P* < 0.05) (Fig. 1D).

### Electrofusion-induced ER stress in rabbit SCNT embryos

The levels of ER stress-associated transcripts, including IRE-1 and CHOP, were measured at the 1-, 2-, 4-, 8-, and 16-cell, morula, and blastocyst stages using real-time PCR (Fig. 1E,F). In the results, there were no significant differences of the expressions of IRE-1 and CHOP in the 1-, 2-, and 4-cell stage embryos among the 3.2 kV/cm SCNT group, 1.6 kV/cm SCNT group and non-electrofusion group. Compared with those in the non-electrofusion group, the expression of IRE-1 and CHOP was markedly increased in the 3.2 kV/cm SCNT group and the 1.6 kV/cm SCNT group at the 8- and 16-cell, morula, and blastocyst stages (*P* < 0.05). Additionally, the expressions of IRE-1 and CHOP in the 1.6 kV/cm SCNT group were significantly lower than that in the 3.2 kV/cm SCNT group (*P* < 0.05).

### Melatonin reduced ROS and ameliorated the abnormal epigenetic state and ER stress in rabbit SCNT embryos

**Figure 2:**
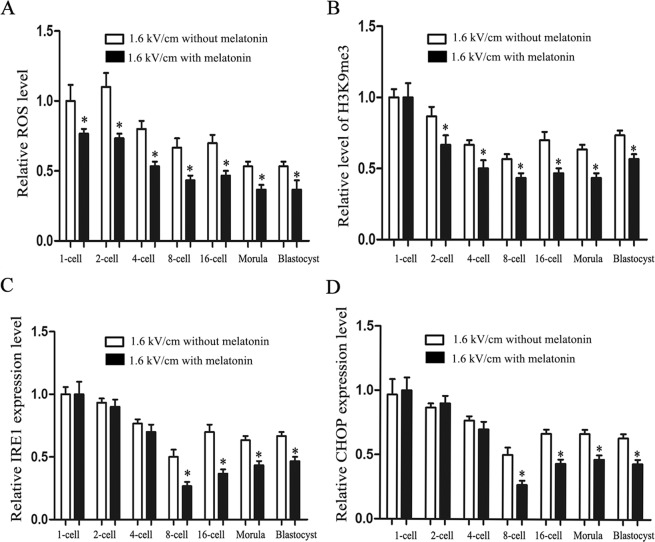
Effects of melatonin on SCNT embryos. (**A**) The quantitation of ROS in rabbit SCNT embryos treated with melatonin at 0 and 10^−6^ M at the indicated stage. (**B**) The effect of electrofusion on H3K9me3 in SCNT embryos treated with melatonin at 0 and 10^−6^ M at the indicated stage. The relative expression levels of IRE1 (**C**) and CHOP (**D**) in rabbit SCNT embryos treated with melatonin at 0 and 10^−6^ M at the indicated stage. (^*^P < 0.05).

To determine the optimal melatonin concentration, SCNT embryos were treated with different concentrations of melatonin throughout the preimplantation development period. The results showed that the blastocyst rate in the 10^−6^ M melatonin group was significantly higher than that in other groups (*P* < 0.05) (Table 2). Compared with the group without melatonin, the group with 10^−6^ M melatonin had significantly reduced ROS throughout the preimplantation stage (*P* < 0.05) (Fig. 2A), and decreased H3K9me3 levels at the 2-, 4-, 8-cell, morula, and blastocyst stages in the SCNT group (*P* < 0.05) (Fig. 2B). The treatment with melatonin also decreased the expression of IRE-1 (*P* < 0.05) (Fig. 2C) and CHOP (*P* < 0.05) (Fig. 2D) at the 8- and 16-cell, morula, and blastocyst stages.

**Table 2:** Reconstructed rate, cleavage rate and blastocyst rate of rabbit cloned embryos at different melatonin concentrations.

Melatonin Concentration	No. oocytes	Reconstructed rate (%)	Cleavage rate (%)	Blastocyst rate (%)
0 M	205	173 (84.4)^a^	135 (78.0)^a^	44 (32.5)^a^
10^−3^ M	202	171 (84.6)^a^	130 (76.0)^a^	43 (33.1)^a^
10^−6^ M	196	165 (84.1)^a^	136 (82.4)^a^	61 (44.9)^b^
10^−9^ M	201	164 (81.6)^a^	129 (78.7)^a^	42 (32.6)^a^

Different superscripts within same column indicate significant difference (P < 0.05).

The parameter was three 20 μsec DC pulses at 1.6 kV/cm.

### Melatonin protects against electrofusion-induced damage in rabbit SCNT embryos

**Figure 3:**
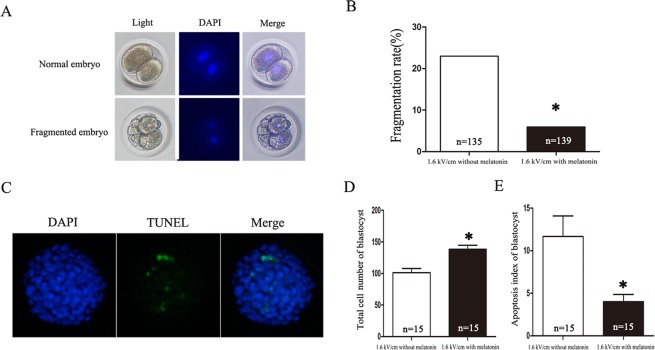
Effects of melatonin on SCNT blastocyst development. (**A**) A bright-field image of four embryos showing normal cleavage or fragmentation. (**B**) The percentage of fragmented embryos after treatment with melatonin at 0 and 10^−6^ M. (**C**) Blastomeres stained by TUNEL assay for apoptosis (green) and DAPI for nuclei (blue). The total number of cells per blastocyst (**D**) with 10^−6^ M melatonin treatment were significantly increased compared to the untreated group. The apoptotic index (**E**) of the group treated with 10^−6^ M melatonin was significantly reduced compared to the untreated group (*P < 0.05). The number of embryos per group is indicated by ‘n’.

The embryos at 1.6 kV/cm electrical stimulation were cultured with or without melatonin (10^−6^ M). The fragmentation rate, the total cell number of blastocysts and the apoptotic index of blastocysts were analyzed. The results showed that the treatment with melatonin significantly reduced the fragmentation rate (*P* < 0.05) (Fig. 3A,B), increased the total cell numbers (*P* < 0.05) (Fig. 3C,D), and decreased the apoptotic index of blastocysts (*P* < 0.05) (Fig. 3C,E).

## Discussion

Up to now, electrofusion was the most commonly used method for most SCNT studies^8,9,18,19^. Regarding the piezo microinjection method, researchers were concerned about the risk of mercury contamination during the injection using micropipettes^20^. The mercury vapor and mercury compounds are highly toxic, so the piezo method is very risky to operators and embryos^21,22^. And also, the electric or chemical activations were required immediately after the establishment of embryos^23^. Here, we determined that the cleavage and blastocyst rates in the non-electrofusion group were significantly lower than in the electrofusion groups, indicating that the electrofusion method was better than the piezo method in terms of activating SCNT embryos. Considering its safety, applicability and better activation, we opted to use the electrofusion procedure and studied its effects on SCNT embryos, oxidative stress, epigenetic state and ER stress.

Electrical stimulation can lead to oxidative stress in myotubule cells via regulating the phosphorylation of AMPK^24^. Rapid, transient electrical stimulation increased the level of ROS in cardiac myocytes. In addition, previous studies showed higher ROS levels in SCNT embryos, which could inhibit embryo development^25^. Antioxidants are often added to culture medium to increase antioxidant capacity in embryo culture^26,27^. Melatonin is an antioxidant and free-radical scavenger that may be beneficial for the production of developmentally competent embryos when electrofusion is employed^5^. Melatonin effectively reduced oxidative stress and improves the development and hatchability rates of preimplantation fertilized embryos and SCNT embryos in cows, pigs, goats, mice and other species^5,18,28,29^. The results in the present study showed that greater electrical stimulation induced higher ROS level in SCNT embryos, which were reduced by incubation with melatonin.

Electromagnetic stimulation can lead to specific activation of the histone acetyltransferase, Brd2, resulting in histone H3K27 acetylation^17^. Electrical stimulation causes rat muscle dysfunction by altering miRNA-related signaling pathways^30^. Also, electrical stimulation modulates the gap junction-mediated cardiac cell-cell communication by regulating histone acetylase and deacetylase activities^16^. Chromatin structures were changed in BlCa cells exposed to ROS, and high levels of ROS increased H3K9me3 expression in human BlCa tissues^31^. H3K9me3 is highly conserved in most animals and is associated with the transcriptional inhibition of euchromatin, repression of chromatin, and formation of heterochromatin and plays a pivotal role in mammalian development^1,32^. The elevated level of H3K9me3a is a major epigenetic obstacle to the process of somatic cell nuclear reprogramming^1^. Our previous study reported that the inhibition of SUV39H1 can downregulate H3K9me3 and improve the efficiency of SCNT^33^. Importantly, embryonic genome activation (EGA) is the most important event during reprogramming, and EGA occurs around the 8-cell stage in rabbit embryos^34–36^. In this study, we found that electrical stimulation increased the level of H3K9me3 in SCNT embryos at the 2-, 4- and 8-cell stages, while stronger electrical stimulation caused H3K9me3 to be expressed at even higher levels. Su *et al*. demonstrated that melatonin has a positive role on modifying the epigenetic state of embryo via scavenging ROS^5^. And in this study, we found that electrofusion changed the epigenetics of SCNT embryos, and these epigenetic changes can be protected by melatonin.

Electrical stimulation such as in the treatment of tumors can induce endoplasmic reticulum (ER) stress and cause cell apoptosis^37^. When cells were subjected to stimulation, the unfolded protein response (UPR) was activated via the PERK, ATF6, and IRE1 pathways^38^. Pro-apoptotic factors, such as the CHOP, ASK1, JNK and Bcl2 protein families, were activated and overexpressed, inducing apoptosis and cell death^38,39^. Electrical stimulation can disrupt intracellular membranes, including those of the ER, mitochondria, and nucleus, thereby inducing apoptotic pathways^39,40^. In this study, we found that there were no significant differences in the expression levels of IRE-1 and CHOP in 1-, 2-, and 4-cell stage embryos among the 3.2 kV/cm SCNT group, 1.6 kV/cm SCNT group and the control group. It was conjectured that before the EGA stage, the embryonic genes were not activated, and the expression of ER stress genes was mainly derived from maternal storage. With the development of the EGA stage, embryonic genes were activated, and gene expression was affected by the epigenetic state of the embryos. We also demonstrated that H3K9me3 expression levels were influenced by electrical stimulation. We found that the expressions of IRE-1 and CHOP were markedly increased in the 3.2 kV/cm SCNT group and the 1.6 kV/cm SCNT group at the 8-cell, 16-cell, morula, and blastocyst stages. The results indicated that the ER stress at the post-EGA stage was regulated by embryonic genes whose control depended on the epigenetic state of the SCNT embryos. Previous studies reported that melatonin improved the meiotic maturation of porcine oocytes by reducing ER stress, and melatonin can also alleviate lipopolysaccharide-induced placental cellular stress response in mice^41,42^. These positive functions were thanks to the inhibition of oxidative stress function of melatonin^41,42^. In the present study, we also found that treatment with melatonin significantly ameliorated SCNT embryo ER stress induced by electrical stimulation.

The fragmentation rate is an important index for assessing the developmental competence of embryo^43^. Abnormal cleavage can cause defects in chromosome segregation, the appearance of haploid cells, and impaired embryonic development^44^. The total cell numbers in blastocysts and the apoptotic index are also important indicators of embryonic developmental competence^45–49^. In this study, we found that incubation with melatonin significantly reduced the fragmentation rate, increased the total cell number in blastocysts and decreased apoptosis, indicating that melatonin protects against electrical stimulation-induced damage in rabbit SCNT embryos. The positive effects of melatonin may be a result of the reduction of ROS, a decrease in ER stress and the preservation of normal epigenetic modifications.

To conclude, we discovered that electrofusion induced oxidative stress, increased H3K9me3, and led to ER stress in rabbit SCNT embryos, while melatonin protected against those changes. Our findings are of signal importance for improving the efficiency of SCNT and for optimizing the application of electrical stimulation in other biomedical areas.

## Material and Methods

### Reagents and media

Unless otherwise stated, culture media and reagents used in this study were purchased from Sigma (St. Louis, MO, USA).

### Animal preparation

The experimental protocol was in accordance with the National Institutes of Health Guide for Care and Use of Laboratory Animals and was approved by the Laboratory Animal Care Committee of Xi’an Jiaotong University. New Zealand white rabbits, approximately 3 kg in weight and 12 months old were used. Rabbits were raised in the Laboratory Animal Center of Xi’an Jiaotong University Health Science Center at a temperature of 24 ± 1 °C, relative humidity of 55 ± 5%, and a 12 h light:12 h dark photoperiod. The animals were given access to water and food *ad libitum*.

### Collection of oocytes

Female rabbits were super-ovulated by subcutaneous injection of 80 IU of pregnant mare serum gonadotropin (PMSG; Sansheng, Ningbo, China) and after 96 h, 100 IU of chorionic gonadotropin (CG; Sansheng, Ningbo, China) was administered by intravenous injection. After 12 h, the rabbits were euthanized and the oviducts and uteri were removed and flushed with 5 mL of Dulbecco’s phosphate-buffered saline (DPBS) to recover the oocytes. The oocytes were observed under a microscope and transferred into DPBS containing 5% fetal bovine serum (FBS).

### Preparation of donor cells

A small piece of ear skin (1 × 1 cm) from a three year-old New Zealand white rabbit was removed, washed three times with DPBS containing penicillin and streptomycin, cut into small pieces, and cultured for approximately 10 days in DMEM containing 10% FBS, penicillin and streptomycin to obtain fibroblasts. The fibroblasts were cultured in saturation humidity with 5% CO_2_ at 38.5 °C. When the cells were approximately 80% confluent, the fibroblasts were subcultured and transferred into a 3.5-cm dish with fresh culture medium. Fibroblasts were purified through two passages. Fibroblasts from 3–4 passages were cultured to confluence in 96-well plates (Corning, NY, USA) and then incubated in DMEM supplemented with 0.5% FBS for 3 days. The cells were detached with DPBS containing 0.25% (w/v) trypsin and 1 mM ethylenediaminetetraacetic acid (EDTA) for 1 min, trypsinization was terminated by addition of DMEM containing 10% FBS, and cells were pelleted by centrifugation at 350 g for 10 min. The pellets were resuspended in M199 containing 7.5 m g/mL cytochalasin B and 10% FBS for SCNT.

### Somatic cell nuclear transfer (SCNT)

SCNT was conducted as previously described^8^. Oocytes containing the first polar body were selected and stained with 7.5 μg/mL Hoechst 33342 in Earle’s balanced salt solution-complete medium (Thermo Fisher Scientific; Waltham, MA, USA) for 20 min. The oocytes were then placed in M199 containing 7.5 μg/mL cytochalasin B for 10 min. The nuclei of oocytes were localized by UV illumination for 1 to 2 s and removed using an 18 μm outer diameter micropipette. For electrofusion, a donor cell was drawn up and injected into the perivitelline space of an enucleated oocyte. The microelectrode consisted of a 25 cm long copper wire with a diameter of 100 microns passed through a 20 cm long plastic tube with an outer diameter 3.5 mm and inner diameter 2.5 mm. The copper wire was fixed to the plastic tube with glue so that electrostatic interference from the plastic tubing and the micromanipulation system and operators could be avoided. The copper wire was soldered with an electric soldering iron to a 5 cm long platinum wire curved at about 135 degrees. A second electrode was made in the same way, and each was attached to a micromanipulator controlled by the micromanipulation system (Eppendorf, Saxony, Germany). The embryos were incubated three times with fusion medium (BTX; Holliston, MA, USA) for 3 min, and then transferred to a fusion medium droplet. The complex was gently clamped with microelectrodes to maintain contact with the cell membrane. The electrofusion conditions were three 20 μs DC pulses at 1.6 kV/cm or 3.2 kV/cm with an ElectroSquare Porator (BTX; ECM830). After fusion, the somatic embryos were washed three times with DPBS and transferred into embryo culture medium for one hour at 38.5 °C, 5% CO_2_, and 100% humidity. For the non-electrofusion group, a donor cell was drawn up and injected into the enucleated oocyte, washed three times with DPBS, transferred into embryo culture medium and incubated for one hour at 38.5 °C, 5% CO_2_, and 100% humidity.

### Embryos activation and culture

The fused embryos or injected embryos were treated with ionomycin for 5 min and then washed three times with DPBS. The embryos were treated for 1 h with 2 mM 6-dimethylaminopurine (6-DMAP) and 5 μg/mL cycloheximide (CHX; Abcam) in Earle’s balanced salt solution (EBSS)-complete medium. The embryos were then washed three times with DPBS. The cloned embryos were subsequently cultured in SOF medium (Caisson, Wuhan, China) containing 6% BSA at 38.5 °C in 5% CO_2_ at 100% humidity. Melatonin was added to the culture medium at concentrations of 0, 10^−3^, 10^−6^, and 10^−9^ M and was present throughout the development period. After 4 h of culture, the reconstructed embryos were examined microscopically and the number of cells counted. We examined the embryos again after 24 hours of culture and counted the number of embryos undergoing cleavage and the number of fragmented embryo. After four days in culture, the numbers of blastocysts were counted, and samples of blastocysts from the pool were collected for the measurement of ROS, immunofluorescence staining of H3K9me3, mRNA expression levels using real-time PCR, apoptosis detection, and counting of total cell numbers per blastocyst. The reconstruction rate was determined by the normalization of the number of reconstructed embryos to that of donor oocytes. The cleavage rate was determined by the normalization of the number of cleavage embryos to that of reconstructed embryos. The fragmentation rate was defined as the ratio of the number of fragmented embryos to that of cleavage embryos. The blastocyst rate was defined as the ratio of the number of blastocysts to the number of cleavage embryos.

### Measurement of reactive oxygen species in embryos

A reactive oxygen species assay kit (Beyotime, Shanghai, China) was used to quantitate the ROS levels. Embryos were incubated in serum-free culture medium containing 10 mM dichlorodihydrofluorescein diacetate (DCHF-DA) at 37 °C for 20 min. The embryos were then washed three times in serum-free culture medium and fluorescence examined using the Nikon Eclipse Ti-S microscope (Nikon, Tokyo, Japan). Images were captured using a digital camera, analyzed with Image-Pro Plus software (Media Cybernetics, Bethesda, MD), and background fluorescence was subtracted from the experimental readings. Experiments were performed in triplicate and fifteen embryos were analyzed for each group at each stage.

### Quantitation of H3K9me3 by immunofluorescence

Embryos (15 per group at each stage) were incubated in pronase and acidic Tyrode’s solution to remove the zona pellucida and mucin coat. Embryos were then fixed in 4% paraformaldehyde for 30 min, permeabilized with 0.1% Triton X-100 for 20 min, and blocked in 1% BSA at 4 °C overnight. Embryos were stained with anti-H3K9me3 antibody (Abcam, Cambridge, MA, USA) at 4 °C overnight, washed thoroughly, and incubated with secondary antibody, Alexa Fluor 488-labeled goat anti-mouse IgG (Beyotime), for 1 h at room temperature. The embryos were counterstained with DAPI (Beyotime), mounted, and fixed with a drop of Antifade Mounting Medium (Beyotime). The immunofluorescent signals of the samples were detected using a laser scanning confocal microscope (Zeiss, Jena, Germany). The mean intensity of fluorescence was measured using Image-Pro Plus 6.0 software (Media Cybernetics). Each nucleus was manually outlined and the mean fluorescence intensities for H3K9me3- and DAPI-stained samples were recorded, and divided by the acquisition times of the corresponding signal. The total fluorescent intensities for H3K9me3 and DAPI were then obtained according to the amount of the embryos.

### Real-time PCR

Total RNA was extracted from embryos using the Cells-to-Signal Kit (Invitrogen, Carlsbad, CA, USA) and the PrimeScript^TM^ RT Reagent Kit (TaKaRa, Tokyo, Japan) was used to synthesize cDNA. Real-time PCR was performed using the CFX96 RT-PCR detection system (Bio-Rad, Hercules, CA, USA) with SYBR Premix Ex Taq^TM^ II (TaKaRa, Tokyo, Japan). The thermocycling conditions were as follows: 1 min at 95 °C, followed by 40 cycles of 5 s at 95 °C and 30 s at 60 °C, and a melting curve ranging from 65 °C to 95 °C, 5 s per 0.5 °C increment. The specificity of the real-time PCR reaction was confirmed by single peaks in the melt curves. Data were collected during each extension. Amplification efficiencies of genes were obtained using the slopes of the standard curves. The geometric mean of the Ct values of H2AFZ and HPTR1 was adopted to normalize the gene expression as previously described. The primers were designed using Primer3 and sequences are shown in Table 3. Twenty embryos per group at each stage were processed in each replication and measurements of gene expression were performed in triplicate.

**Table 3:** Primer list.

Gene	Sense primer (5′–3′)	Antisense primer (5′–3′)
HPTR1	ACGTCGAGGACTTGGAAAGGGTGTT	GGCCTCCCATCTCCTTCATCACATC
H2AFZ	AGAGCCGGCTGCCAGTTCC	CAGTCGCGCCCACACGTCC
IRE1	ACCTAGTGAGCTGTGCGTCC	GGGGATGCCTGTACCAACTC
CHOP	TTGCCTTTCTCCTTCGGGAC	TCCAGGGGGTGAGACATAGG

### Apoptosis measurement and counting of total cells per blastocyst

The DeadEnd Fluorometric TUNEL System (Promega, Madison, WI) was used according to the manufacturer’s instructions to quantitate apoptosis. Blastocysts (15 per group) were fixed in 4% paraformaldehyde at room temperature for 2 h, permeabilized in 0.5% Triton X-100 at room temperature for 5 min, and incubated with FITC-conjugated dUTP and terminal deoxynucleotidyl transferase in the dark at 37 °C for 1 h. The tailing reaction was terminated by addition of 2 × SSC in the dark for 15 min at room temperature. The embryos were then incubated with PBS containing 25 µg/mL RNase A in the dark at room temperature for 30 min. After DAPI staining and washing with PBS in the dark, the blastocysts were placed on a slide, mounted with a coverslip, and examined using the Nikon Eclipse Ti-S fluorescence microscope. Images were captured with a digital camera and analyzed using Image-Pro Plus software. The total number of cells per blastocyst was indicated by DAPI staining.

### Statistical analysis

Statistical analysis was conducted using the SPSS software package (SPSS Inc., Chicago, IL, USA). The reconstructed rate, the cleavage rate, the fragmentation rate, and the blastocyst rate were analyzed using χ^2^ tests. The relative intense of H3K9me3, apoptotic index, and total cell numbers were analyzed using one-way ANOVA. The equal variance was analyzed by the Levene median test and ANOVA, and multiple pairwise comparisons were performed by Tukey’s test. A *P* value less than 0.05 (*P* < 0.05) was considered statistically significant. Error bars on the means represent ± SEM.

## Data Availability

The datasets generated and analyzed during the current study are available from the corresponding author on reasonable request.
